# Saguenay Youth Study: A multi-generational approach to studying virtual trajectories of the brain and cardio-metabolic health

**DOI:** 10.1016/j.dcn.2014.10.003

**Published:** 2014-10-23

**Authors:** T. Paus, Z. Pausova, M. Abrahamowicz, D. Gaudet, G. Leonard, G.B. Pike, L. Richer

**Affiliations:** a Rotman Research Institute, University of Toronto, Toronto, Canada; b Hospital for Sick Children, University of Toronto, Toronto, Canada; c McGill University Health Centre, McGill University, Montreal, Canada; d Community Genomic Medicine Centre, Department of Medicine, Université de Montréal, Chicoutimi, Canada; e Montreal Neurological Institute, McGill University, Montreal, Canada; f Hotchkiss Brain Institute, University of Calgary, Calgary, Canada; g Department of Health Sciences, University of Quebec in Chicoutimi, Chicoutimi, Canada

**Keywords:** Adolescence, Middle age, MRI, Brain, Mental health, Addiction

## Abstract

This paper provides an overview of the Saguenay Youth Study (SYS) and its parental arm. The overarching goal of this effort is to develop trans-generational models of developmental cascades contributing to the emergence of common chronic disorders, such as depression, addictions, dementia and cardio-metabolic diseases. Over the past 10 years, we have acquired detailed brain and cardio-metabolic phenotypes, and genome-wide genotypes, in 1029 adolescents recruited in a population with a known genetic founder effect. At present, we are extending this dataset to acquire comparable phenotypes and genotypes in the biological parents of these individuals. After providing conceptual background for this work (transactions across time, systems and organs), we describe briefly the tools employed in the adolescent arm of this cohort and highlight some of the initial accomplishments. We then outline in detail the phenotyping protocol used to acquire comparable data in the parents.

## Highlights

• Psychiatric conditions contribute the most to global health burden.
• Developmental cascades provide a framework for understanding causes and consequences.
• Multi-generational design enables studies of virtual health trajectories.
• Saguenay Youth Study is a multi-generational and multi-system cohort designed along these lines.

## 1 Introduction

The last century witnessed a dramatic growth in life expectancy. In the Unites States of America, life expectancy increased from an average of 44.8/47.8 (men/women) years in 1900 to an average of 73.9/79.4 years in 1998 (Smith and Bradshaw, 2006), owing mainly to the development of treatments of infectious diseases and the management of cardiovascular disorders and cancers (Guyer et al., 2000). Unfortunately, this increase in life expectancy has not been paralleled by increases in healthy life expectancy, defined as years lived without a disability. In 2002, the global (194 countries) gap between life expectancy and health-adjusted life expectancy was 7.5 years (Mathers et al., 2004). In developed countries, the main causes of Years Lived with Disability (YLD) – a metric used to calculate health-adjusted life expectancy – are non-communicable diseases (86.2% of all causes), with psychiatric conditions contributing the most (41.9%) to the overall health burden (Mathers et al., 2004). Among the latter conditions, unipolar depressive disorder (15%), alcohol abuse (6.8%) and Alzheimer's disease and other dementias (4.2%) stand out as the major causes of YLD. Although the increased prevalence of dementias is in part a reflection of longer life span, a cumulative impact of poor cardio-metabolic health on brain health is also one of the key mechanistic pathways leading to dementia (see below).

**Fig. 1:**
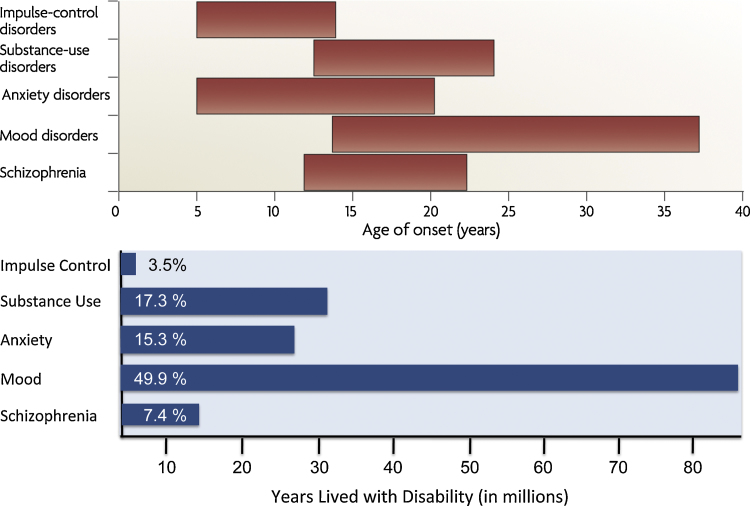
Ranges of onset age (top) and years lived with disability (bottom) for common psychiatric disorders. Top figure adapted from (Paus et al., 2008a). Bottom figure based on data from (Whiteford et al., 2013). Percentages indicate proportion of years lived with disability explained by each mental and substance use disorder group in 2010 (100% = all psychiatric disorders).

One of the main reasons for the high health-burden associated with psychiatric disorders, such as depression and substance use, but also schizophrenia (2.3%) and bipolar disorder (2.2%), is their early onset and chronic course, resulting in a large accumulation of YLD over time (Fig. 1).

For this reason, our quest to understand the causes and pathways leading to psychiatric disorders must take a developmental perspective. This perspective acknowledges the complexity of developmental cascades – and ensuing trans*actions* – playing out over time, across levels and between organs (Masten and Cicchetti, 2010). In the following text, we will review briefly these three elements of developmental cascades in order to provide context for the design of the Saguenay Youth Study (SYS). We will conclude this section by providing motivation for expanding the SYS to include a multi-generational arm.

### 1.1 Developmental cascades and transactions

**Fig. 2:**
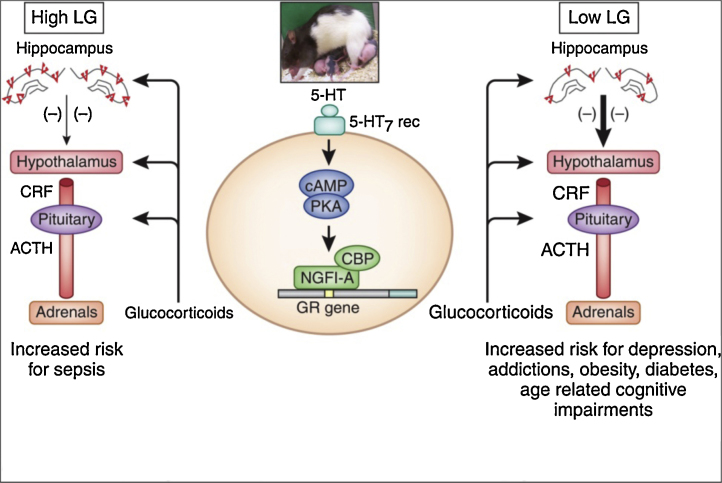
Associations between maternal behavior (licking and grooming), expression of the glucocorticoid receptor in the hippocampus, regulation of the hypothalamus-pituitary-adrenal axis and psychopathology (right side). From (Zhang et al., 2013). LG, licking and grooming; ACTH, adrenocorticotropin; CRF, corticotropin releasing factor; 5-HT, serotonin; camp, cyclic adenosine monophosphate; PKA, protein kinase A; NGFI-A, nerve growth factor-inducible factor A; CBP, CREB-binding protein; GR, glucocorticoid receptor.

1.1.1. *Transactions over time* is perhaps the best-understood long-term driver of health: a cascade starting in pregnancy and early post-natal development, progressing through “preclinical” stages of a disease (e.g., in adolescence), and ending in a disease with its fully expressed manifestations (e.g., in adulthood). Let us illustrate this concept with two seminal studies of the relationship between early environment and adult health. In experimental settings, Meaney and colleagues have demonstrated that maternal care (licking and grooming of pups) has a striking impact on the hypothalamus-pituitary-adrenal axis (HPA) and stress reactivity of the offspring (Liu et al., 1997). This, in turn, sets in motion a number of pathophysiological processes leading ultimately to poor mental and physical health (Fig. 2). Using an epidemiological approach, Barker discovered an association between birth weight and cardiovascular mortality in adulthood (Barker et al., 1989). Here, a cascade initiated in utero and in early post-natal life may exert a domino effect on cardio-metabolic health of the offspring for the rest of his/her life (Plagemann, 2006). These are two examples of early “programming” of the brain and body systems, with powerful long-term consequences for mental and cardio-metabolic health. Not surprisingly, many scholars interested in long-term consequences of early adversity have embraced the hypothesis of developmental origins of disease (Wadhwa et al., 2009). Given this perspective, longitudinal studies of health trajectories are clearly the most suitable approach for investigating developmental cascades over time, though the complexity of such cascades requires large samples. Although a number of birth cohorts (∼831 to 100,000 participants each) have been studying antecedents of mental health in a prospective fashion, a recent review suggests that many of these cohorts fall short due to the limited “breadth and depth of measurement” necessary for enhancing our understanding of “how pre- and perinatal factors and early neurodevelopment relate to child psychopathology” (Thompson et al., 2010). From the perspective of the developmental cognitive neuroscientist, the use of magnetic resonance imaging (MRI) for quantifying brain development provides both the breadth and depth of quantitative phenotypes relevant for mental health. In this regard, the Generation R Study stands out as the only birth cohort that has begun serial MR scanning of its members. Out of the total of 7893 Generation-R children, 1000 of them have been scanned between 6 and 8 years of age and 5000 children will be scanned between 10 and 12 years of age (White et al., 2013).

1.1.2. *Transactions across levels* (molecules, physiological systems, individual behavior, and social groups) are critical for our understanding of pathways underlying given health trajectories. The above example of maternal care (licking and grooming) and HPA reactivity illustrates cogently this perspective (Zhang et al., 2013). As shown in Fig. 2, this cascade begins with an environmental manipulation – maternal behavior – measured at the behavioral level (licking and grooming behaviors), but its effect on the offspring HPA system can be assessed at a systems level (circulating levels of glucocorticoids), and the molecular level (expression of glucocorticoid receptors in the hippocampus). And consequences on offspring behavior can be assessed at the behavioral level (self-administration of cocaine).

This example from experimental work carried out in rodents highlights one of the challenges of the human work: the absence of direct observations of behavior in large epidemiological studies – whether in the context of “exposures” (e.g., maternal behavior) or “outcomes” (i.e., offspring behavior). Instead, we rely on self-reports by the parents and the offspring. On the other hand, MR imaging provides a rich source of quantitative phenotypes that can be used to characterize the state of structural and functional organization of the offspring brain at a system level (phenomics). In addition, carefully designed cognitive batteries can provide system level assessments of key processes underlying a given behavior (e.g., decision-making or reward sensitivity tested in the laboratory). Finally, a sample of blood (or saliva) can be the source of biological material for both the system level (e.g., stress and sex hormones) and molecular-level (e.g., genetic [genomics] and epigenetic [epigenomics] variations) assessments. We have reviewed the use of the different “omics” sciences in population-based studies elsewhere (Paus, 2013).

**Fig. 3:**
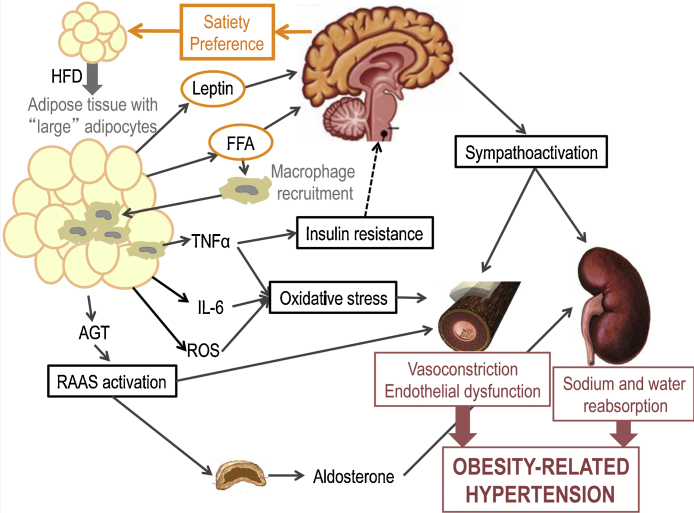
Transactions across organs: from eating behavior, through fat cells to cardiovascular regulation. Based on (Pausova, 2006). From (Paus, 2013). HFD, high-fat diet; FFA, free fatty acids; AGT, angiotensinogen; TNFα, tumor necrosis factor alpha; IL-6, interleukin 6; ROS, reactive oxygen species; RAAS, rennin-angiotensin-aldosterone system.

1.1.3. *Transactions across organs* highlight the importance of an integrated approach to mental and physical health. As pointed out above, Alzheimer's disease (AD) is an excellent example of the interplay between cardio-metabolic and brain health. Over 50% of the population attributable risk of AD is modifiable: diabetes, midlife hypertension, midlife obesity, smoking, depression, cognitive inactivity or low educational attainment, and physical inactivity (Barnes and Yaffe, 2011). As shown in Fig. 3, transactions take place across various “organs”: from fat tissue to the endocrine system (e.g., insulin signaling) and low-grade inflammation (e.g., cytokines) to sympatho-activation, kidney function, blood pressure and cerebro-vascular reactivity, all leading eventually to the deterioration of brain perfusion and metabolism, loss of gray and white-matter and cognitive decline (Paus, 2013).

Furthermore, early environment – and therefore transactions over time – may play an important role even in the case of AD; not only in terms of metabolic programming mentioned above but also vis-à-vis early brain development and, in turn, formation of cognitive and mental-health “reserves” (Stern, 2012). A life-course approach is therefore warranted not only in the case of neuro-developmental disorders, such as depression and addictions, but also in the case of neuro-degenerative disorders, such as AD (Miller and O’Callaghan, 2008).

### 1.2 Virtual health trajectories

Given the need for a life-span approach in studying health trajectories, birth cohorts are a logical solution. Without doubt, such cohorts are invaluable sources of knowledge about developmental cascades. But as pointed out above, very few birth cohorts have the necessary depth of phenotyping (Thompson et al., 2010). Many of the cutting-edge tools available today did not exist when the original birth cohorts were initiated. At the same time, longevity of phenotyping tools is inevitably shorter than that of the human life span: what is state-of-the-art today may be obsolete (or unavailable) tomorrow.

The *trans-generational* approach represents a possible “short cut” to achieving (and validating) long-term models of health trajectories, while overcoming the challenges associated with implementing and sustaining long-term longitudinal studies. In a multi-generational study, the successive generations represent stages of disease trajectories. In other words, the trans-generational commonalities – based on shared genes (25–50% in a 3-generation family vs. ∼1% in general population) and the shared family environment (e.g., geographical location, lifestyle) – become a “signature” of a given family on which non-shared elements (e.g., individual lifestyle, treatments) operate. By comparing individuals of the same age, but coming from families with different “signatures,” we may be in a position to identify long-term predictors of brain and cardio-metabolic health.

**Fig. 4:**
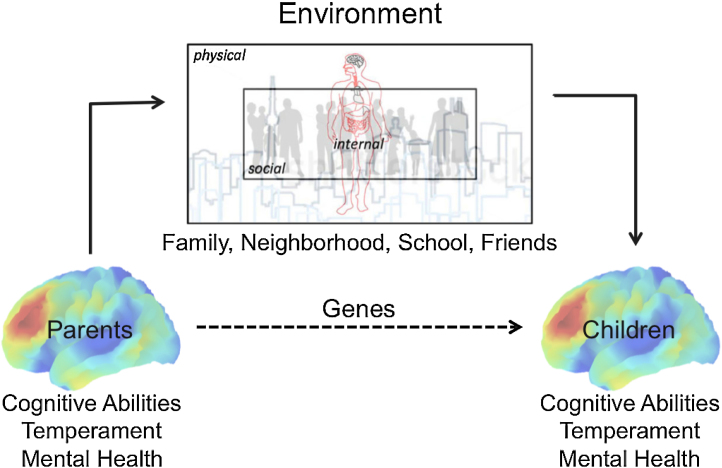
Shaping the child's brain. This schematic diagram illustrates how parents’ brains contribute to the variations in their children's environments – internal (e.g., nutrition), physical (e.g., pollution, noise, access to parks) and social (neighborhood safety, school friends and role models).

We have hypothesized that the accuracy of discriminating between a descendant (e.g., daughter or granddaughter) who will develop a disease and one who will not, tested against the profile (and/or disease status) of her ancestor (e.g., mother or grandmother), will be comparable to the discriminative accuracy observed in recent epidemiological studies but on a shorter time-scale (Paus, 2013). This hypothesis is based on two general observations: (1) most complex traits (and diseases) are likely caused by a host of factors: multiple genes, various environmental influences and, of course, combinations of the two; and (2) genetic and environmental factors cluster in families, further enhancing similarity in complex traits across generations (Fig. 4).

**Fig. 5:**
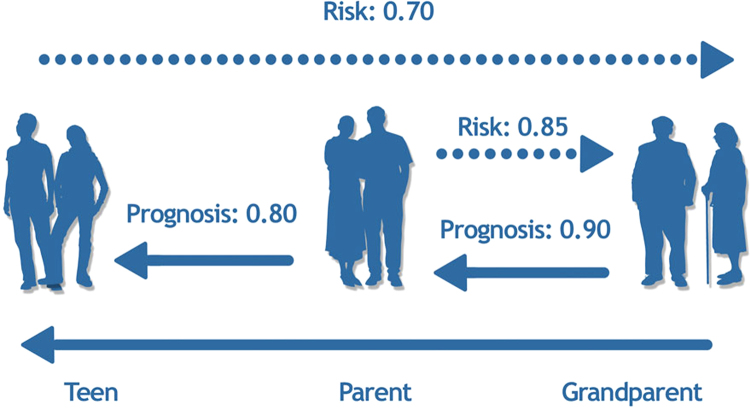
Risk/resilience profiling: virtual validation (dashed lines) and prognosis by ancestor/pedigree (solid lines). The numbers indicate hypothesized discriminative accuracy (0.5 = chance, 1 = perfect discrimination).

In the case of mortality risk, as shown by others from the Rotterdam Study (Hofman et al., 2011), the predictive value of a number of lifestyle and physiological characteristics (162 variables in total) is high vis-à-vis the short-term (<1 year) prediction of mortality (∼0.80) and it decreases to ∼0.70 when death occurs 15 years post-assessment (Walter et al., 2012); this is illustrated by the dashed lines in Fig. 5. If our hypothesis is correct, then Prognosis by Ancestor/Pedigree may be equal to, or better than, a long-term (30+ years) prognosis by one's current health status (Fig. 5, solid lines). If so, Prognosis by Ancestor/Pedigree will provide a glimpse into the future for descendants, as well as providing the basis for specific interventions and preventive measures, so as to avoid predicted adverse outcomes.

From (Paus, 2013).

## 2 Saguenay Youth Study: overall design

The first wave of the SYS cohort (2003–2012) has focused on establishing a community-based sample of adolescents (12 to 18 years of age) in which to evaluate associations between the exposure to an adverse prenatal environment, brain development and cardio-metabolic health (Pausova et al., 2007). In keeping with the above principles of developmental cascades, the cohort was set up so that detailed information could be collected at different levels (behavioral, systemic, molecular) and organs (brain, adipose tissue, cardiovascular system, endocrine system). Over a period of 10 years, we have collected a rich dataset in 1029 adolescents from the Saguenay Lac Saint Jean region (Quebec, Canada). This region is the home of the largest population with a known genetic founder-effect in North America (De Braekeleer, 1991, De Braekeleer et al., 1998, Gradie et al., 1988, Grompe et al., 1994), making it particularly suitable for studies of complex traits.

As a model of prenatal adversity, we chose maternal cigarette smoking during pregnancy (MSP). This choice reflects high prevalence of MSP in the general population; the latest results of the National Survey on Drug Use and Health (U.S.A.) suggest that smoking during pregnancy has not changed significantly between 2002/2003 and 2011/2012, ranging between 18% and 15.9% respectively (Anon., 2013). Smoking during pregnancy has been associated with a number of behavioral sequelae (Cornelius and Day, 2009, Gaysina et al., 2013, Kandel et al., 2009, Wakschlag et al., 2011), in that offspring of mothers who smoked cigarettes during pregnancy are more vulnerable to developing addictions, likely due to the combination of prenatal exposure with other familial (genetic and environmental) risks.

We recruited adolescents in high schools. Over a period of 10 years, our team has made 28 visits to schools and, in this way, contacted a total of 27,190 students (18,127 families). Of the 18,127 families, 5570 (33%) sent a response card indicating their interest in the study (3269 families; 59% of all responses) or declining further participation (2301 families; 41% of all responses). Based on the inclusion (e.g., maternal smoking during pregnancy, 2 or more siblings per family) and exclusion (MR contraindications) criteria (Supplementary Table I in Pausova et al., 2007), a total of 1801 families (55% of the interested families) were eligible to participate in the study; the eligibility was determined by a research nurse via a structured telephone interview. Individuals whose mothers smoked during pregnancy (at least 1 cigarette per day during the second trimester) were identified first (via a telephone interview with the mother); then we selected non-exposed individuals (no smoking for 12 months before pregnancy and during pregnancy) who were matched to the exposed ones by maternal education and school attended. Note that this matching procedure was used on an ongoing basis throughout the study; it was applied at each high school so that an equivalent number of “exposed” and (matched) non-exposed adolescents were recruited at each school. In this manner, we minimized differences between the “exposed” and “non-exposed” adolescents in their families’ socio-economic status. We used a family-based design, recruiting a minimum of two siblings per family; note that siblings were concordant for the exposure status in the majority of families (446/481 families; 93%). Phenotyping of the adolescents took place over several sessions (∼15 h in total) and included a number of domains detailed in Table 1 (further details in Pausova et al., 2007 and www.saguenay-youth-study.org); each adolescent provided a fasting (morning) blood sample.

**Table 1:** Saguenay Youth Study: Baseline in adolescence (completed).

Domain	Tool	Phenotypes
Brain	MRI	Global and regional volumes; cortical surface & thickness; MTR
Cognition	6-h battery	FSIQ, VIQ, PIQ; verbal, visuospatial, working memory; executive functioning, problem solving, fluency, language, phonological and motor skills; social cognition
Mental health	DPS, GRIP	Epidemiological diagnoses; symptom counts
Substance use	GRIPado	Cigarette smoking, cannabis, alcohol use, drug experimentation (age of initiation, last 30-days, binge drinking)
Personality	NEO-PI-R	Neuroticism, extroversion, openness, agreeableness, conscientiousness
Sexual maturation	PDS	Stages of pubertal development (Tanner stages)
Lifestyle	Lerner, 24-h food recall	Sleep, energy and nutrient intake, physical activity, extracurricular activities, sexuality, academic/vocational aspirations
Family environment	FamEnvi	Stressful life events, financial difficulties, SES (family income, parental education)
Body composition	Anthropometry, MRI, Bioimpedance	Height, weight, circumferences, skinfolds; subcutaneous, visceral fat and muscle volumes; fat & muscle mass
Cardiovascular	Finometer	Beat-by-beat blood pressure and heart rate at rest and in response to physical and mental challenges, sympathetic & parasympathetic tone
Hormones	Blood	Testosterone, estrogen, cortisol
Biochemistry	Blood	Glucose, insulin, cholesterol, HDL-cholesterol, triglycerides, leptin, C-reactive protein, glycerol, free fatty acids
Lipidomics	LC-ESI-MS	∼700 lipid species

MTR, magnetization transfer ratio; DPS, DISC Predictive Scales; GRIP, Groupe de Recherche sur l’Inadaptation Psychosociale, adolescent self-assessment of mental health and substance use developed for the SYS by J. Séguin based on validated National Longitudinal Survey of Children and Youth (NLSCY) and Quebec Longitudinal Study of Child Development (QLSCD) protocols; Lerner, adolescent self-assessment developed by Richard Lerner. FSIQ Full_Scale IQ Rating; VIQ, Verbal IQ Rating; PIQ, Performance IQ Rating; PDS, Puberty Development Scale; HDL, high-density lipoprotein; LC-ESI-MS, liquid-chromatography electrospray-ionization mass-spectrometry; NEO-PI, Neuroticism, Extraversion, Openness–Personality Inventory. For details, see Pausova et al., 2007.

During this initial phase (Wave 1), biological parents of the adolescents filled out a series of questionnaires about the family environment and their mental health; the latter included questions about cigarette smoking, alcohol use, and drug experimentation throughout their life (including current habits and age of onset), and the presence of anti-social behavior (at present and during their adolescence; Table 2). Parents also provided a blood sample for genetic analyses.

**Table 2:** Saguenay Youth Study: Baseline in parents (completed).

Domain	Tool	Phenotypes
Family environment	FamEnvi	Stressful life events, financial difficulties, SES (family income, parental education)
Mental health	GRIPadult	Symptom counts (depression, anxiety, anti-social behavior)
Substance use	GRIPadult	Cigarette smoking, alcohol use, drug experimentation

FamEnvi, questionnaire on family environment developed by the SYS team; GRIPAdult, self-assessment of mental health and substance use, as adapted by colleagues at the Groupe de Recherche sur l’Inadaptation Psychosociale of the University of Montreal.

In Table 3, we provide basic demographic information about the sample of adolescents who underwent the full assessment during Wave 1. In Table 4, we provide information about the lifetime history and current (last 30 days) use of the three most common substances: cannabis, alcohol and cigarettes. Note that the current use of cannabis in older adolescents (16–18 years) is comparable to that found in other countries among high-school students (Hibell et al., 2012).

**Table 3:** Wave 1: Baseline in adolescents (completed).

Measure	Distribution
*N*	1029
Number of families	481
Age (years)	Mean = 15.02; SD = 1.84
Sex	48% male; 52% female
Exposure to MSP	48% exposed; 52% non-exposed
Household income	≤$20,000 – 13% $30,000–40,000 – 19% $50,000–60,000 – 24% $70,000–80,000 – 20% ≥$85,000 – 24%
Full scale IQ	Mean = 104.42; SD = 12.14

SD = Standard deviation; IQ = intelligence quotient; MSP = maternal smoking during pregnancy.

**Table 4:** Percent of adolescents reporting drug experimentation (lifetime) and current use (last 30 days) for cannabis, alcohol and cigarettes in the SYS adolescents.

	*N*	Cannabis (lifetime)	Cannabis (last 30 days)	Alcohol (lifetime)	Alcohol (last 30 days)	Cigarettes (lifetime)	Cigarettes (last 30 days)
Early adolescence (12–15.9 years)	704	22%	9%	52%	21%	21%	8%
Late adolescence (16–18 years)	324	55%	20%	91%	65%	38%	22%

Using deoxyribonucleic acid (DNA) extracted from the blood samples of the adolescents and their parents, we have acquired information about single nucleotide polymorphisms (SNPs) using a genome-wide approach. The first 600 adolescents were genotyped with the Illumina Human610-Quad BeadChip (610K SNPs). The remaining 424 adolescents and all 971 parents were genotyped with the Illumina HumanOmniExpress BeadChip (700K SNPs). We have used imputations to generate the same set of markers for the two samples; we employed an imputation protocol developed by the ENIGMA Working Group, and imputed genotypes using IMPUTE (www.mathgen.stats.ox.ac.uk/impute), with a reference file created by the ENIGMA2 Genetics Support Team. This reference file is based on the most recent versions of the 1000 Genomes Project set (Phase 1, Release v3; ∼41M SNPs) but includes only ∼13M SNPs that are polymorphic in Caucasians and have been observed more than once in European populations.

In addition, we have quantified the rate of DNA methylations across 450,000 CpG sites in a subset of the adolescents (*n* = 132) and their parents (*n* = 280); this was accomplished by hybridizing DNA to the Infinium HumanMethylation450 BeadChip (Illumina, San Diego, CA). This chip interrogates methylation at >485,000 CpG sites, providing coverage of >99% RefSeq genes; the CpG sites are targeted across gene regions including the promoter, 5′UTR, first exon, gene body, and 3′UTR, as well as intergenic sequences (Sandoval et al., 2011).

Finally, targeted lipidomics profiling is currently conducted in all adolescents and their parents. Liquid chromatography, electrospray ionization mass spectrometry (LC-ESI-MS) is used to assess plasma profiles of >700 glycerolipid, glycerophospholipid and sphingolipid species as potential biomarkers of cardiovascular and mental health.

## 3 Saguenay Youth Study: highlights

Before proceeding with the description of the parent arm of the SYS cohort (Wave 2: Parents), let us briefly highlight some of the published observations made in Wave 1: Adolescents. We will focus here on findings relevant to maternal smoking during pregnancy and addictive behavior; our work on sex differences in the maturation of white matter (Herve et al., 2009, Perrin et al., 2008, Perrin et al., 2009), puberty-related changes in the face morphology (Mareckova et al., 2011, Mareckova et al., 2013) and cardio-metabolic health (Goodwin et al., 2013, Melka et al., 2013a, Melka et al., 2012, Pausova et al., 2010, Pausova et al., 2012, Syme et al., 2008, Syme et al., 2009) can be found elsewhere.

**Fig. 6:**
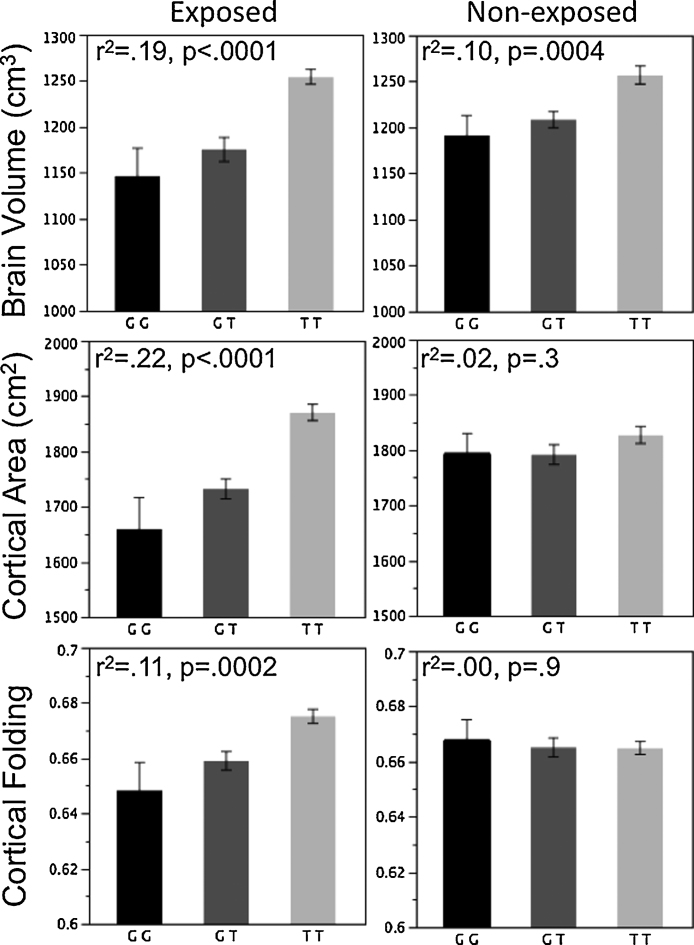
Age-adjusted values (Mean ± SE) of brain volume (top), total cortical area (middle) and cortical folding (bottom) in female adolescents exposed (left column) and not exposed (right column) to maternal cigarette smoking during pregnancy, all plotted as a function of the *KCTD8* genotype (rs716890; GG, GT and TT genotypes; G, guanine; T, thymine). The amount of variance (*r*^2^) explained by the genotype and statistical significance of the genotype effect of each phenotype are indicated. Note that we observed significant interaction between the *KCTD8* genotype (rs716890) and “exposure” on total cortical area and cortical folding but not on brain volume.

One of the most common consequences of MSP is intra-uterine growth retardation (Lowe, 1959); this is not surprising given the multiple effects of cigarette smoking on the supply of nutrients and oxygen to the fetus (reviewed in Pausova et al., 2007, Slotkin, 1998). As shown with fetal imaging, brain growth does not appear to escape this global phenomenon (Anblagan et al., 2013). By the time the exposed offspring reaches adolescence, however, the brain size appears to be the same as that of non-exposed adolescents. Nonetheless, we asked whether this is the case also for individuals with a particular genetic variation associated with brain size, as revealed in a genome-wide association study (GWAS) in the SYS adolescents. We found that this was not so: exposed female adolescents with the *KCTD8* risk-variant had smaller surface area of the cerebral cortex than non-exposed females without this variant (Fig. 6; Paus et al., 2012). We have speculated that this gene-environment interaction reflects an accelerated apoptosis of progenitor cells in the developing brains of embryos/fetuses who possess this particular genetic variant (Paus et al., 2012). Above and beyond global brain growth, we have observed differences between the exposed and non-exposed (female) adolescents in the (relative) size of the corpus callosum (Paus et al., 2008b) and the thickness of the orbitofrontal cortex, OFC (Toro et al., 2008). We followed up the latter finding and asked whether there is a relationship between the OFC thickness and drug experimentation; in this context, we have examined the role of the known functional polymorphism in the *BDNF* gene (and the methylation status of its promoters) in moderating this relationship (Lotfipour et al., 2009, Toledo-Rodriguez et al., 2010). We also investigated a relationship between genetic variations in alpha 6 nicotinic receptor gene (*CHRNA6*), striatal volume and drug experimentation (Lotfipour et al., 2010).

From (Paus et al., 2012).

Prenatal exposure to maternal smoking during pregnancy is a well-established risk factor for obesity (Al Mamun et al., 2006, Ino, 2010, Leary et al., 2006, Oken et al., 2005, Oken et al., 2008, Power et al., 2010, Power and Jefferis, 2002, Syme et al., 2010, von Kries et al., 2002, Weng et al., 2012). In an exposed individual, it increases the likelihood for developing obesity by 50%, (Ino, 2010, Weng et al., 2012). Given this higher risk (odds ratio of 1.5), and the prevalence of MSP in the 1960s and 1970s (40%) and currently (16%), we estimated that – at present – up to 16% of obesity in middle-aged adults and 7.4% of obesity in children is attributable to MSP. The underlying mechanisms of the link between MSP and obesity are not clear, however. Our findings in the SYS suggest that reward-related mechanisms may be at play. We showed that MSP is associated with substantial increases in body adiposity (Syme et al., 2010), and higher preference for fat accompanied by smaller amygdalae volumes (Haghighi et al., 2013). In a genome-wide association study, we also showed that dietary preference for fat (as well as body adiposity) is associated with genetic variation in the opioid receptor mu 1 gene (*OPRM1*) (Haghighi et al., 2014). Finally, we have demonstrated that MSP is associated with modifications of DNA methylation that persist into adolescence of the exposed offspring (Lee et al., 2014a), and that some of these modifications are present in *OPRM1*, and may inhibit expression of the protective (fat intake-lowering) allele of this gene (Lee et al., 2014b). Taken together, these observations suggest (a) the presence of relationships between the brain-reward system, dietary preference for fat and obesity; (b) perturbations of these relationships by MSP and genetic variations in *OPRM1*; and (c) DNA methylation as a possible molecular mechanism underlying interactions between environment (MSP) and genes (*OPRM1*).

Apart from the above fat-preference angle, we also found that a genetic variant of the *FTO* gene, the best known genetic risk factor for obesity, predicts an inverse relationship between total brain volume and fat body mass (Melka et al., 2013b). We have suggested that, given the completion of overall brain growth in early childhood, these effects might have their origins during early development and reflect a differential commitment of progenitor cells to ectoderm (brain) or mesoderm (fat tissue). Finally, we began exploring the differential impact of visceral fat on the adolescent's brain (Schwartz et al., 2014) and cognition (Schwartz et al., 2013).

Overall, the initial findings obtained in Wave 1 of the SYS cohort revealed a number of associations between MSP, brain, and addictive behavior of adolescents in the context of both drug experimentation and dietary fat preference. Although observational studies cannot attribute solely such associations to MSP (D’Onofrio et al., 2012), human studies with genetically sensitive research designs (Gaysina et al., 2013) and preclinical studies of prenatal exposure to nicotine (Franke et al., 2008) suggest that these prenatal exposures do play a role in shaping mental-health trajectories. Furthermore, careful consideration of potential confounders, such as maternal education and alcohol use during pregnancy, when examining the relations between externalizing behavior and substance use (Lotfipour et al., 2014) or the specificity of some of the observed structure–function relationships (e.g., correlation between the amygdala volume with fat preference but not with alcohol use; Haghighi et al., 2013) suggest that these associations are not due to global phenomena (such as “poverty” or an “addictive personality” of the parents). Finally, it is important to note that we have observed a number of gene-exposure interactions that argue against general confounding: with the equal distribution of a given genetic variant between the “exposed” and “non-exposed” adolescents, such gene–environment interactions point to specific molecular pathways mediating associations between MSP and a given phenotype (*CHRNA6* (Lotfipour et al., 2010); *KCTD8* (Paus et al., 2012); *OPMR1* (Lee et al., 2014a)). In general, the use of genetic variations to ascribe causality to observations made in large epidemiological studies has been helpful in a number of domains (Smith and Ebrahim, 2003). In the context of addiction, we employ this approach in secondary analyses carried out by the members of a consortium called “The Causal Analysis Research in Tobacco and Alcohol (CARTA)”, which includes 30 studies, with a total of 150,000 participants (http://goo.gl/dkXQyH).

## 4 Saguenay Youth Study: parent arm

As pointed out above, we have obtained basic information about the mental health and substance use of the biological parents at the time of recruitment of the adolescents, as well as a blood sample for genetic and epigenetic analyses. In Table 5, Table 6, respectively, we provide summaries of demographic information about the parents, their mental health and substance use at the time of the initial recruitment (Wave 1).

**Table 5:** Wave 1: Baseline in parents (completed).

Measure	Distribution
*N*	962
Number of families	481
Age (years)	Mean = 43.33; SD = 4.58
Sex	50% male; 50% female
Household income	≤$20,000 – 13% $30,000–40,000 – 19% $50,000–60,000 – 24% $70,000–80,000 – 20% ≥$85,000 – 24%
Education	No high school – 1% Some high school – 15% High school – 52% College degree – 19% Bachelors – 9% Masters or doctorate – 3% Unknown – 1%

SD = standard deviation.

**Table 6:** Wave 1: Parents. Substance use and mental health.

	Mothers (%)	Fathers (%)
Smoking (current/former/never)	32/42/26	31/41/29
Cannabis (last 12 months)	5.2	11.2
Alcohol (binge drinking^a^)	49.8	71.15
Depression symptoms (90th percentile)	10.6	9.7
Anxiety symptoms (90th percentile)	12.5	6.9

a 5 or more drinks on one occasion (at least once in the last 12 months).

In 2012, we initiated Wave 2: Parents of the SYS cohort. This wave focuses on deep phenotyping of the biological parents of the SYS adolescents. Parents complete a series of on-line questionnaires and visit our phenotyping unit for MRI scans of the brain and abdominal fat, blood sample for biochemistry and lipidomics analyses, as well as for cardio-metabolic and cognitive assessments (Table 7). The visit lasts ∼4 h.

**Table 7:** Wave 2: parents (ongoing).

Domain	Tool	Phenotypes
Brain	MRI	Global and regional volumes; cortical surface & thickness; white-matter hyperintensities; magnetization transfer ratio; diffusion tensor imaging; resting-state functional MRI
Cognition	Cambridge Brain Sciences Platform	Executive functioning; attention; learning & memory; reasoning; spatial skills
Mental health	MINI International Neuropsychiatric Interview; Mental Health and Addiction Questionnaire; ASR; CES-D; Family History Screen	Depression, anxiety, attention deficit hyperactive disorder, antisocial personality disorder; post-traumatic stress disorder; obsessive compulsive disorder; alcohol and substance dependence, bulimia, anorexia; family history of psychiatric disorders
Substance use & addiction	Mental Health and Addiction Questionnaire; YFAS; FNDS; AUDIT; SRE; ESPAD; IAT; SOGS	Cigarette smoking, alcohol and drug use, gambling, internet addition, food addiction
Personality	NEO-FFI	Neuroticism, extroversion, openness, agreeableness, conscientiousness
Lifestyle	Life Experiences Questionnaire; PBI; Hand Preference	Family characteristics; education; socio-economic status; physical activity; sexual activity; parental style; hand laterality
Sleep	PSQI; ESS	Sleep quality, latency, duration, efficiency and disturbances; daytime sleepiness
Body composition	Anthropometry, MRI, Bioimpedance	Height, weight, circumferences, skinfolds; subcutaneous, visceral fat and muscle volumes; fat & muscle mass
Cardiovascular	Finometer	Beat-by-beat blood pressure and heart rate at rest and in response to physical and mental challenges, sympathetic & parasympathetic tone
Lung Function	Spirometer	Forced vital capacity, forced expiratory volume
Diet	24-h food recall	Energy and nutrient intake
Medical history	Medical Questionnaire	Personal and family history of: cancer, hypertension, diabetes, heart disease, lipid disease, psychiatric disorders, addiction; reproductive and sexual health; medications
Hormones	Blood	Testosterone, estrogen, cortisol
Biochemistry	Blood	Lipid profile (TG, TC, HDL-C, LDL-C), glucose, insulin, free fatty acids, glycerol, C-reactive protein
Lipidomics	LC-ESI-MS (Blood)	>700 lipid species

MRI = magnetic resonance imaging; Cambridge Brain Sciences Platform (Hampshire, Highfield, Parkin, & Owen, 2012); MINI International Neuropsychiatric Interview (Sheehan et al., 1998); Mental Health and Addiction Questionnaire (Adapted from the Ontario Health Study www.ontariohealthstudy.ca and the Wave-1 questionnaire developed by the SYS team); ASR = Adult Self Report (Achenbach & Rescorla, 2003); CES-D = Center for Epidemiology Studies Depression Scale (Radloff, 1977); Family History Screen (Weissman et al., 2000); YFAS = Yale Food Addiction Scale (Gearhardt et al., 2009); FNDS = Fagerström's Nicotine Dependence Scale (Heatherton et al., 1991); AUDIT = Alcohol Use Disorder Identification Test (Barbor et al., 1992); SRE = Subjective Response to Ethanol (Schuckit et al., 1997); ESPAD = European School Survey Project on Alcohol and Other Drugs (Hibell et al., 2012); IAT = Internet Addiction Test (Widyanto and McMurran, 2004); SOGS = South Oaks Gambling Screen (Lesieur & Blume, 1987); NEO-FFI = NEO-Five Factor Inventory (Costa and McCrae, 1992); Life Experiences Questionnaire (Adapted from the Ontario Health Study www.ontariohealthstudy.ca and the Wave-1 questionnaire developed by the SYS team); PBI = Parental Bonding Instrument (Parker et al., 1979); Hand Preference (Adapted from Crovitz and Zener (1962)); PSQI = Pittsburgh Sleep Quality Index (Buysse et al., 1989); ESS = Epsworth Sleepiness Scale (Johns, 1991); 24-h Food Recall (Buzzard et al., 1996); Medical Questionnaire (Adapted from the Ontario Health Study www.ontariohealthstudy.ca); TG = triglycerides; TC = Total Cholesterol; HDL-C = High density lipoprotein-cholesterol; LDL-C = low density lipoprotein-cholesterol; LC-ESI-MS = liquid chromatography electrospray ionization mass spectrometry.

### 4.1 Internet-based assessments

As indicated in Table 7, we are re-administering family environment, lifestyle and mental-health/substance use questionnaires, as well as several new questionnaires focusing on parenting, sleep, personality and various addictive behaviors, such as food, gambling and internet addictions.

### 4.2 Face-to-face assessment

The visit takes place in the morning and lasts ∼4 h: it includes a draw of blood (after overnight fasting) for future “omics” analyses, a structured psychiatric interview, cognitive assessment, MRI and cardiovascular/body-composition sessions, each lasting ∼60 min. For the *psychiatric interview*, we use the Mini-International Psychiatric Interview (MINI Plus, Sheehan et al., 1998) administered by a trained research assistant. MINI Plus is a validated, structured psychiatric interview for current and lifetime DSM-IV and ICD-10 psychiatric disorders. We have also added the nicotine dependence module from Semi-Structured Assessment for the Genetics of Alcoholism, SSAGA (Hesselbrock et al., 1999).

### 4.3 Cognitive abilities

*Cognitive abilities* are assessed using a validated web-based battery for the assessment of cognition developed by Drs. Owen and Hampshire (www.cambridgebrainscience.com). The battery is comprised of 12 computer-based tests of executive function, memory, learning and attention, and takes ∼35 min to complete. Population norms are available from two large-scale public trials involving more than 100,000 participants (Hampshire et al., 2012).

### 4.4 The MRI session

*The MRI session* takes place at a private MR clinic in Chicoutimi, which is equipped with a Siemens 1.5T (Avanto) scanner. A 60-min MR session includes the following imaging protocols. *Structural MRI of the brain*: T1W, 1-mm, isotropic images acquired with a 3D fast RF-spoiled gradient echo scan. *Magnetization-transfer* (*MT*) *ratio:* MT data are acquired using a dual acquisition (3D RF-spoiled gradient echo scan) with and without an MT saturation pulse (3-mm thick axial slices, 1 × 1 mm in-plane resolution). *Diffusion Tensor Imaging* (*DTI*): DTI is used to assess the structural properties of white matter. Diffusion encoding is achieved using a single-shot, spin-echo, echo planar sequence with twice-refocused balanced diffusion encoding gradients (64 diffusion-encoding directions, 3-mm thick axial slices, 2.3 mm × 2.3 mm in-plane resolution). *Resting-state functional MRI* is acquired over a period of 12 min (260 volumes, 4-mm thick axial slices, 3.5 mm × 3.5 mm in-plane resolution, eyes closed). Finally, abdominal scans are acquired using heavily T1-weighted, spin-echo scans (30 axial 10-mm thick slices, 1-mm gap, 0.9 mm × 0.9 mm in-plane resolution) extending from about the bottom of the liver to the umbilicus level.

### 4.5 The cardiovascular/body composition session

This session takes place at ECOGENE-21/Community Genomic Medicine Center in Chicoutimi. The following protocol is identical to the one used to collect equivalent data in adolescents during Wave 1. *Blood pressure* (BP) is measured both under standard clinical conditions and during a 52-min cardiovascular reactivity protocol. (a) *Standard clinical conditions:* BP is measured at rest for at least 10 min while seated with a standard occlusion cuff (“The fourth report on the diagnosis, evaluation, and treatment of high blood pressure in children and adolescents,” 2004). This measurement is done at the beginning and end of the cardiovascular reactivity protocol described next. (b) *Cardiovascular reactivity protocol* lasts 52 min and mimics daily-life activities, including changes in posture and mental stress; BP and a number of other cardiovascular parameters are measured beat-by-beat using a non-invasive hemodynamic monitor, Finometer (FMS Finapres, Amsterdam, The Netherlands; see below). The posture test consists of 3 periods during which the participant rests in a supine position for 10 min, stands for 10 min, and sits for 10 min. The mental stress test involves a 30-s explanation administered 5 min prior to a 2-min sequence of 23 simple arithmetic problems, each presented for 5 s; the problems include simple math additions or subtractions followed by simple multiplications or divisions. The level of difficulty increases progressively with time to ensure some failures for all participants; all answers are recorded. The math sequence is followed by a 10-min period of resting in a sitting position. Throughout this protocol, a Finometer is used to record continuously the finger blood flow. The Finometer derives beat-by-beat brachial systolic and diastolic BP from the reconstructed and level-corrected finger blood-flow waveform. The Finometer is a reliable device for tracking BP in adults and children older than six years (Parati et al., 1989, Tanaka et al., 1994) and the precision of BP measurement with this device meets the requirements of the American Association for the Advancement of Medical Instruments (Guelen et al., 2008, Westerhof et al., 2002). *Body Composition*: Body weight, height, six circumferences (upper arm, waist, hips, proximal thigh, middle thigh and distal thigh), and five skinfolds (triceps, biceps, subscapular, suprailiac and mid-thigh) are measured according to standard procedures (Pausova et al., 2001). Bioelectrical impedance is used to measure total body fat, total body water and fat-free mass. Participants are asked to refrain from caffeine, alcohol, and vigorous activity 24 h before the test. The actual measurement is made after a 20-min stabilization period during which the participants rest in a supine position. These measures are complemented by the quantification of subcutaneous and visceral fat derived from abdominal MRIs (see above). Given the high prevalence of smokers in this cohort (due to the ascertainment of the original sample, with half of the mothers smoking during pregnancy), we have also included assessment of lung function using *spirometry*. Using a spirometer (MiniSpir by Medical International Research [MIR], Rome, Italy (www.spirometry.com); MiniSpir User's Manual. Manual revision 1.3. 2006. Medical International Research; User's Manual Code 980255) we measure the amount and the rate at which a participant exhales in a single breath. The standard spirometric test requires the participant to exhale as forcefully as possible after taking a full inspiration. This measures the participant's forced vital capacity as well as their forced expiratory volume in one second.

As of September 2014, we have acquired full datasets in 573 parents. We are also in the process of contacting the living grandparents of the adolescents in order to obtain a saliva sample (for genetic and epigenetic analyses) and basic information about their mental health and substance use. Finally, we have obtained approval for receiving information contained in death certificates for the deceased parents and grandparents of the SYS adolescents.

## 5 Large developmental cohorts: design strategies and challenges

The Saguenay Youth Study (and its parental arm) is one among a growing number of large community-based studies (IMAGEN (Schumann et al., 2010); Generation R (White et al., 2013); PING (Fjell et al., 2012)) that combine brain imaging with genetics, as well as with a detailed assessment of cognition, mental health and family environment. The overarching goal of these studies is to gain insights into factors (and mechanisms) shaping the brain in typically developing children and adolescents. These studies face similar challenges with regards to the ascertainment of their participants (and representativeness of the samples), the choice of neuroimaging protocols (structural vs. functional), assessments of cognition and mental health, and involvement of other family members. We will address briefly some of these issues in the following text (for details, see Paus, 2010, Paus, 2013).

Ideally, ascertainment of participants in population-based studies should be free of selection biases, thus creating conditions for generating data representative of the general population – “a representative brain” (Falk et al., 2013). As we pointed out elsewhere, previous imaging cohorts used different recruitment strategies (samples of convenience vs. census-based sampling) and exclusion criteria (MR contraindications only vs. screening out children with any personal and family-based risk factors); not surprisingly, some of these strategies yielded “supernormal” samples (Paus, 2010). In the Saguenay Youth Study, we have carried out recruitment in all public high schools in the region and excluded from participation only adolescents with MRI contraindications and serious conditions likely to affect the brain (e.g., epilepsy) or heart (e.g., heart defects) development. By design, the sample is enriched by individuals born to mothers smoking cigarettes during pregnancy (50% vs. ∼20% expected in general population). Other – more subtle – biases include the requirement of having siblings and being able to contact both biological parents. The latter conditions are, however, unlikely to reduce representativeness of the sample, as the mean number of children per family in the general population is 1.5 (Quebec, 2014), and the two-parent requirement did not demand cohabitation. Recent replications of the relationship between externalizing behavior and substance use during adolescence in two geographically and culturally distinct samples recruited using very different strategies, namely the Saguenay Youth Study and the Northern Finland Birth Cohort 1986, suggest that findings obtained in our sample are generalizable (Lotfipour et al., 2014).

Brain imaging represents a unique tool allowing one to obtain a wide array of quantitative phenotypes (Table 8). A number of considerations are at play when choosing specific MR sequences. For example, studies of typically developing children and adolescents are more likely to include scans sensitive to changes in myelination (e.g., magnetization transfer ratio or myelin water fraction, Dean et al., 2014) rather than those sensitive to white-matter hyperintensities, which are more common in the aging brain (e.g., T2 fluid attenuated inversion recovery [FLAIR]). But perhaps the most relevant considerations relate to the trait (vs. state) qualities of a given measure; after all, we base most of our developmental work on the assumption that genes and early environments shape brain function and structure in a stable and long-term manner. Therefore, test–retest reliability of imaging-derived measures is paramount. Not surprisingly, various metrics derived from (multi-modal) structural images show high test–retest reliability. For example, Wonderlick and colleagues (Wonderlick et al., 2009) have evaluated test–retest reliability (two sessions, 2 weeks apart), and the influence of several acquisition parameters (same 3T scanner), for a number of morphometric measures derived from T1-weighted images by FreeSurfer. They found that the reliability – estimated with intra-class correlation coefficients (ICCs) – was “excellent” for most measures; with the exception the globus pallidus, all ICCs values were above 0.95. The test-retest reliability of DTI-based measures appears to vary across the measures and fiber tracts. For example, Wang et al. (2012) found excellent reliability (ICCs > 0.75) for the mean length of the corpus callosum and the uncinate fasciculus, and fair reliabilities (ICCs between 0.4 and 0.75) for fractional anisotropy in most fiber tracts. On the other hand, test–retest reliability of data obtained with fMRI has been characterized as “fair” (ICC: 0.4–0.75) in adults and adolescents, and “poor” (ICC < 0.4) in children; it is lower in regions with weak “activation”, as revealed by group t-maps (Caceres et al., 2009, Koolschijn et al., 2011, Plichta et al., 2012). The relatively low test–retest reliability of functional data is likely related to a number of factors, including the fact that the fMRI signal is an indirect measure of brain activity, its measurement is affected by a number of noise-generating factors (e.g., head motion, physiological “noise” related to respiration and cardiac cycle) and, most importantly, by the state of the participant during scanning. The latter factors, such as inter-individual and session-by-session variations in task-related behavior (performance, attention) and general state of arousal (anxiety, sleepiness) are very difficult to assess and control, thereby adding significant error to the measurement of the functional phenotype. For the above reasons, we advocate imaging protocols that put emphasis on multi-modal imaging of brain structure. We consider brain structure “a window into the individual's life history”, a notion supported by the wealth of imaging data on experience-related brain plasticity (Lovden et al., 2013).

**Table 8:** An example of a 60-min MR protocol enabling one to characterize a number of structural and functional properties of the human brain. From Paus, 2013.

MRI sequence	Time (min)	Structure and physiology
T1-weighted	10	Volumes, thickness, folding, shape, tissue density
T2-weighted	4	White matter hyperintensities (number, volume, location)
Diffusion tensor imaging	12	Fractional anisotropy, mean diffusivity, track delineation
Magnetization transfer	8	Myelination index
Arterial spin labeling	5	Perfusion
Resting state functional	8	Spontaneous cerebral networks; functional connectivity
Paradigm-based functional	6–10	Brain response associated with specific stimuli/tasks; functional connectivity

Brain imaging (and genetic) data alone are insufficient for tracking trajectories in brain development. A significant time commitment must be made to the assessment of cognition and mental health. In the Saguenay Youth Study, for example, we have spent ∼8 h of the participant's time in these domains. Given a large variety of approaches and available tools, only broad recommendations can be made. When assessing cognition, we have used a combination of standardized (e.g., WISC-III) and computer-based (e.g., auditory processing) tools combined in two 3-h sessions (adolescents). In adults, we have decided to use a 1-h (standardized) battery of cognitive tests. In the mental-health domain, we have focused on self-reported symptoms rather than using a diagnosis-driven (psychiatric-interview based) approach. Conceptually, this strategy is consistent with a move away from categorical definitions of psychiatric disorders, as defined in the Diagnostic and Statistical Manual of Mental Disorders (DSM), and toward symptoms as a preferred level of analysis (Borsboom et al., 2011). From a methodological standpoint, self-reports are a rich source of reliable information, especially in the context of substance use (Sobell and Sobell, 1990).

Finally, any developmental study must consider the caregivers. As illustrated in Fig. 4, parents are the main source of genetic and environmental influences on the developing brain. For this reason, we have obtained parental DNA and basic information about the mental health of the parents (including anti-social behavior during their adolescence) in Wave 1 of the Saguenay Youth Study, and have embarked on deep phenotyping of the parents in Wave 2. Parents are also a key source of information about the life events encountered by their children at different stages of development; prospective longitudinal birth cohorts, such as the Avon Longitudinal Study of Parents and Children (Boyd et al., 2013), Northern Finland Birth Cohort 1986 (Taanila et al., 2004) or Generation R (Jaddoe et al., 2010) are in an advantageous position to use such information to predict the state of brain development (and mental health) at later point in the lives of their participants.

## 6 Conclusions

As pointed out in Introduction (Section 1.1), the life-span perspective on health trajectories reflects the concept of developmental cascades: trans*actions* occurring over time, as well as across systems and organs. The Saguenay Youth Study and its parent arm attempts to integrate detailed information about brain and cardio-metabolic health acquired at the system and molecular levels in a family-based multi-generational context. We hope that the richness of the dataset will allow us to contribute toward current efforts aimed at distinguishing the key processes of healthy trajectories from those leading to common chronic disorders of the brain and body.

## Conflict of interest

None of the authors has any actual or potential conflict of interest including any financial, personal or other relationships with other people or organizations within three years of beginning the submitted work that could inappropriately influence, or be perceived to influence, their work.
